# Overcoming the radial learning curve in the cath lab: the African journey

**Published:** 2016

In addition to its sessions tailored to the needs of specialists, AfricaPCR also includes workshops specifically designed for allied health professionals. One of the highlights of this year’s programme was a lively interactive debate around the pros, cons, challenges and benefits of introducing a radial programme in African cath labs. The meeting was facilitated by registered nurse Sr Diane Kerrigan, radiographer Kerry Moir, and cardiologists Dr Mark Abelson (South Africa) and Dr Jacques Monsegu (France).

Accessing the coronary arteries via the radial artery has been increasing in popularity over the past approximately two decades, as an alternative to the femoral and brachial routes. The first radial interventional programme in South Africa commenced more than 10 years ago. While the left radial artery is usually used for bypass grafting procedures, the right radial is the preferred route for angiography and percutaneous coronary intervention.

Patient selection is highly dependent on operator experience, so repetition of the procedure is key. ‘The more you practise, the better you get’, said Sr Kerrigan.

There are many good reasons for going the radial route. The procedure is more comfortable for the patient. The artery is close to the surface and has a dual blood supply. It’s associated with fewer renal, and nerve and vascular complications. Transfusion requirements are less. Reduced staff utilisation translates into reduced hospital-related costs, as does a shorter hospital stay. Patients can be mobilised earlier. Radial access is also associated with lower mortality rates than with the femoral access.

However, for radial access to be undertaken successfully, it is important for the cath lab team to be ready. It requires trained allied professionals, lots of experience, a supportive, quiet and relaxed environment, and good communication. It is also imperative that all the correct equipment be available. Sr Kerrigan highlighted the four key areas/learning steps necessary to ensure a successful radial programme:

preparation and comfort of the patient, including sedation
accessing the radial artery
management of complications
haemostasis

As previously noted, using the right radial artery is the default practice in South Africa, but there might be a case for using the left radial as it is a more direct route to the coronary arteries. Also, left radial access is more similar to femoral access, something that may be an advantage in the earlier stages of the radial learning curve. Offsetting this, however, is that using the right radial is more comfortable for the cardiologist and exposes the patient to less radiation.

Summing up, Sr Kerrigan underscored these take-home messages. ‘Be aware of the learning curve. Make sure the patient has a good pulse and is comfortable. Use the appropriate cocktail of medications. Choose the catheter carefully and ensure good haemostasis.’

